# Eviprostat Activates cAMP Signaling Pathway and Suppresses Bladder Smooth Muscle Cell Proliferation

**DOI:** 10.3390/ijms140612107

**Published:** 2013-06-06

**Authors:** Kai Li, Jian Yao, Yuan Chi, Norifumi Sawada, Isao Araki, Masanori Kitamura, Masayuki Takeda

**Affiliations:** 1 Department of Oncology, First Affiliated Hospital, China Medical University, Shenyang 110001, China; 2 Department of Molecular Signaling, Interdisciplinary Graduate School of Medicine and Engineering, University of Yamanashi, Chuo, Yamanashi 409-3898, Japan; E-Mails: g10sm006@yamanashi.ac.jp (Y.C.); masanori@yamanashi.ac.jp (M.K.); 3 Department of Urology, Interdisciplinary Graduate School of Medicine and Engineering, University of Yamanashi, Chuo, Yamanashi 409-3898, Japan; E-Mails: nsawada1970@gmail.com (N.S.); matakeda@yamanashi.ac.jp (M.T.); 4 Department of Urology, Shiga University of Medical Science, Ohtsu, Shiga 520-2192, Japan; E-Mail: iaraki@belle.shiga-med.ac.jp

**Keywords:** Eviprostat, cAMP signaling, PDGF, cell proliferation, mesangial cells

## Abstract

Eviprostat is a popular phytotherapeutic agent for the treatment of lower urinary tract symptoms (LUTS). At present, the signaling mechanisms underlying its therapeutic effects are still poorly understood. Given that cAMP has been reported to suppress cell hyperplasia and hypertrophy in various pathological situations, we asked whether the effect of Eviprostat could be ascribed to the activation of the cAMP signaling pathway. In the study, exposure of cAMP response element (CRE)-secreted alkaline phosphatase (SEAP) (CRE-SEAP)-reporter cells to Eviprostat elevated SEAP secretion, which was associated with an increased phosphorylation of vasodilator-stimulated phosphoprotein (VASP) and cAMP-response element-binding protein (CREB), as well as enhanced expression of CRE-regulated protein connexin43, indicating an activation of the cAMP signaling pathway. Consistent with these observations, Eviprostat-induced expression of Cx43 was abolished in the presence of adenylyl cyclase inhibitor SQ22536 or PKA inhibitor H89, whereas it was mimicked by adenylyl cyclase activator, forskolin. Further analysis demonstrated that Eviprostat significantly potentiated the effect of phosphodiesterase 3 (PDE3) inhibitor, but not that of PDE4 inhibitor, on CRE activation. Moreover, Eviprostat suppressed PDGF-induced activation of ERK and Akt and inhibited cell proliferation and hillock formation in both mesangial cells and bladder smooth muscle cells. Collectively, activation of the cAMP signaling pathway could be an important mechanism by which Eviprostat exerts its therapeutic effects for LUTS.

## 1. Introduction

Benign prostatic hyperplasia (BPH) is a common disease in older men, frequently accompanied by prostatic inflammation [1,2]. It has been reported that prostatic inflammation plays a role in the induction and progression of BPH. BPH leads to bladder outlet obstruction (BOO), which is associated with obvious changes in bladder structure and function characterized by lower urinary tract symptoms (LUTS), such as urinary frequency and urgency.

Eviprostat, a plant extract, has widely been used clinically for the treatment of BPH and LUTS in Germany and Japan. Eviprostat consists of five components: extracts from *Chimaphila umbellata*, *Populus tremula*, *Pulsatilla pratensis* and *Equisetum arvense* and germ oil from *Triticum aestivum*. Previous studies have revealed that Eviprostat improved voiding function in LUTS patients and exerted anti-inflammatory and anti-oxidative effects in both clinical and basic experiments [3,4]. For example, a basic study reported that a component of Eviprostat, *Equisetum arvense*, prevented inflammatory responses in a rat model with carrageenan-induced paw edema [5]. However, because Eviprostat is a complex mixture of compounds from multiple natural sources, the exact molecular mechanisms of its beneficial effects remain to be clarified.

In cellular response to inflammation, cAMP is regarded as an important immunomodulator. Through activation of PKA, cAMP is involved in regulating multiple downstream signaling pathways and providing a strong inhibitory signal for inflammatory reaction [6]. Many anti-inflammation drugs have been shown to exert therapeutic effect through activation of cAMP signaling. A non-specific cAMP elevating agent produced from plant (*Coleus forskohlii*), forskolin (FSK), and its derivatives have been reported to be able to inhibit T-cell proliferation and activation, as well as proinflammatory cytokine production [6]. Besides suppression of inflammatory responses, cAMP has also been reported to suppress cell proliferation and hypertrophy in a variety of pathological situations [7,8]. It also has anti-oxidative effects [9]. Because of the similarity between the reported effects of cAMP and those of Eviprostat, we wondered whether the therapeutic effects of Eviprostat could be mediated by activation of the cAMP signaling pathway. The main purpose of this study is to test this hypothesis.

In LUTS caused by BOO, multiple growth factors have been implicated in bladder adaption and structural remodeling [10,11]. We have reported that platelet-derived growth factor (PDGF) plays an important role in the process [12]. PDGF and its receptors in the bladder are elevated under mechanical strain and contribute to the increased thickness of bladder wall in BOO. It also regulates cell proliferation, migration and survival in smooth muscle cells. Most of the effects of PDGF are mediated by the phosphatidylinositol 3-kinase (PI3K) and mitogen-activated protein kinase (MAPK) pathway [13,14]. As cAMP suppresses cell proliferation in various cell types, we also tested the potential anti-proliferative effect of Eviprostat in bladder smooth muscle cells (BSMCs).

## 2. Results and Discussion

### 2.1. Eviprostat Activates cAMP Signaling Pathway

**Figure 1:**
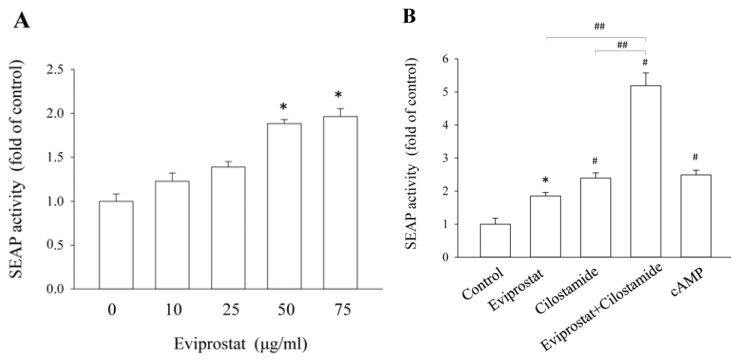

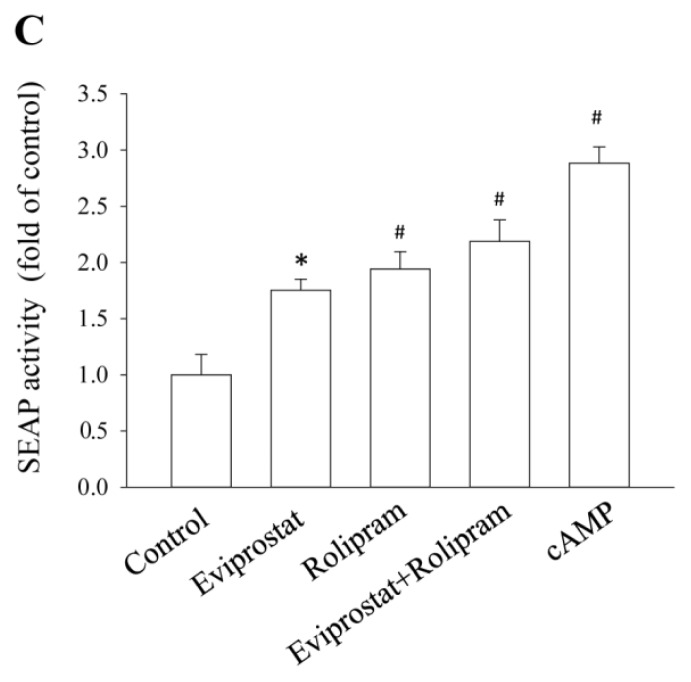
Activation of cAMP response element (CRE)-secreted alkaline phosphatase (SEAP) (CRE-SEAP) by Eviprostat. (**A**) Effects of Eviprostat on CRE activation. Reporter mesangial cells were exposed to the indicated concentration of Eviprostat or 0.1% dimethylsulfoxide, which was used as vehicle control, for 24 h. The conditioned media were harvested and assayed for SEAP activity. Results were expressed as relative induction, compared with the basal level (mean ± SE, *n* = 4), * *p* < 0.05. (**B**,**C**) Effect of Eviprostat on phosphodiesterase 3 (PDE3) or 4 (PDE4) inhibitor-triggered activation of CRE. The cells were treated with 50 μg/mL Eviprostat, 200 μM cAMP, 20 μM cilostamide or 20 μM rolipram alone or in combination for 24 h. ## *p* < 0.01 *vs.* Eviprostat or cilostamide single and * *p* < 0.05, # *p* < 0.01 compared with the control (mean ± SE, *n* = 4).

Because of the similarity between the effects of cAMP and those of Eviprostat on various cellular processes [5,6,15], we tested the possible activation of cAMP signaling pathway by Eviprostat. For this purpose, we have used a well-characterized cAMP response element (CRE)-secreted alkaline phosphatase (SEAP) (CRE-SEAP)-based reporting system [16–18], in which reporter mesangial cells were stably transfected with a vector encoding the reporter gene secretory alkaline phosphatase under the control of CRE. As shown in Figure 1A, incubation of reporter cells with Eviprostat resulted in a concentration-dependent elevation in SEAP activity, indicating an activation of the cAMP signaling pathway.

Our previous study characterized that phosphodiesterase 3 (PDE3) and 4 (PDE4) were two major enzymes for cAMP degradation in the established reporter cells and that the synergistic induction of CRE-SEAP could only be induced by concomitant stimulation of cells with PDE3 and PDE4 inhibitors, but not other PDE inhibitors [16–18]. Taking advantage of this feature, we tested the possible influence of Eviprostat on PDE3 or PDE4. As shown in Figure 1B, Eviprostat potentiated the effect of PDE3 inhibitor cilostamide on the SEAP secretion, but it did not affect the effect of PDE4 inhibitor, rolipram (Figure 1C). These data thus indicate that Eviprostat might activate the cAMP signaling pathway through suppression of PDE4.

**Figure 2:**
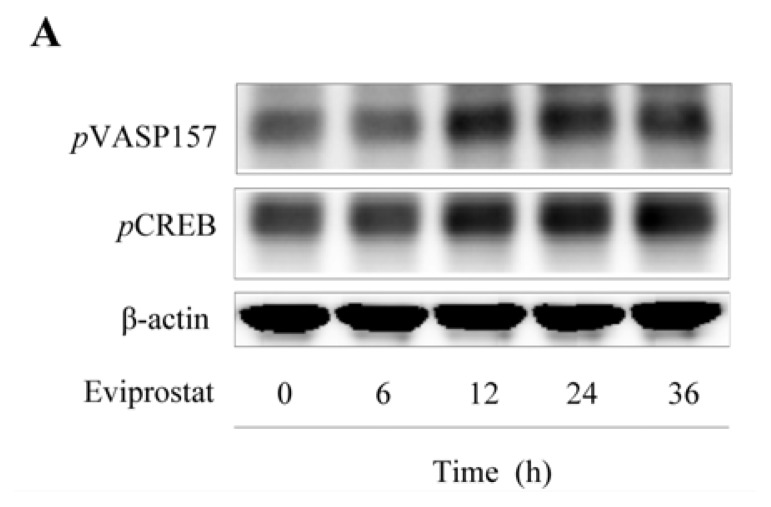

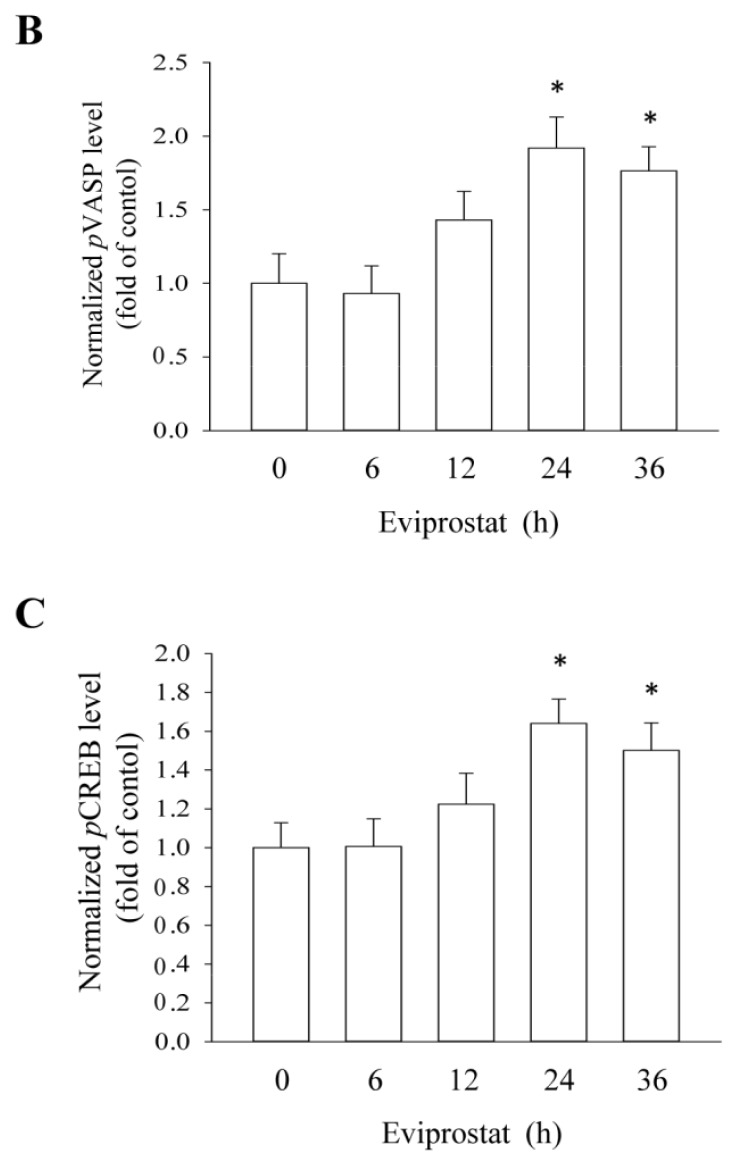
Activation of protein kinase A (PKA) by Eviprostat. (**A**) Time-dependent effects of Eviprostat on phosphorylation of the vasodilator-stimulated phosphoprotein (pVASP) at serine 157 and CREB. Mesangial cells were exposed to 50 μg/mL Eviprostat or 0.1% dimethylsulfoxide as vehicle control for indicated duration Cellular protein was extracted and subjected to Western blot analysis using specific antibodies for *p*VASP and *p*CREB. Equal loading of protein per lane was verified by reprobing the blot with β-actin for loading control. (**B** and **C**) Densitometric analysis of *p*VASP and *p*CREB in A were shown, respectively. The results were normalized to corresponding internal controls and data were presented as induction relative to the basal level in control (mean ± SE, *n* = 4). # *p* < 0.01 *vs.* control.

To further confirm the cAMP-activating effect of Eviprostat, we examined the phosphorylation levels of VASP, a validated substrate of cAMP-dependent protein kinase A (PKA) [19]. As shown in Figure 2, Eviprostat induced a time-dependent increase in the level of VASP phosphorylation at serine 157 (Figure 2B), indicating an activation of PKA. Consistently, Eviprostat also induced CREB phosphorylation, another well characterized PKA substrate (Figure 2C).

### 2.2. Eviprostat Elevates Cx43 Expression via cAMP Signaling Pathway

**Figure 3:**
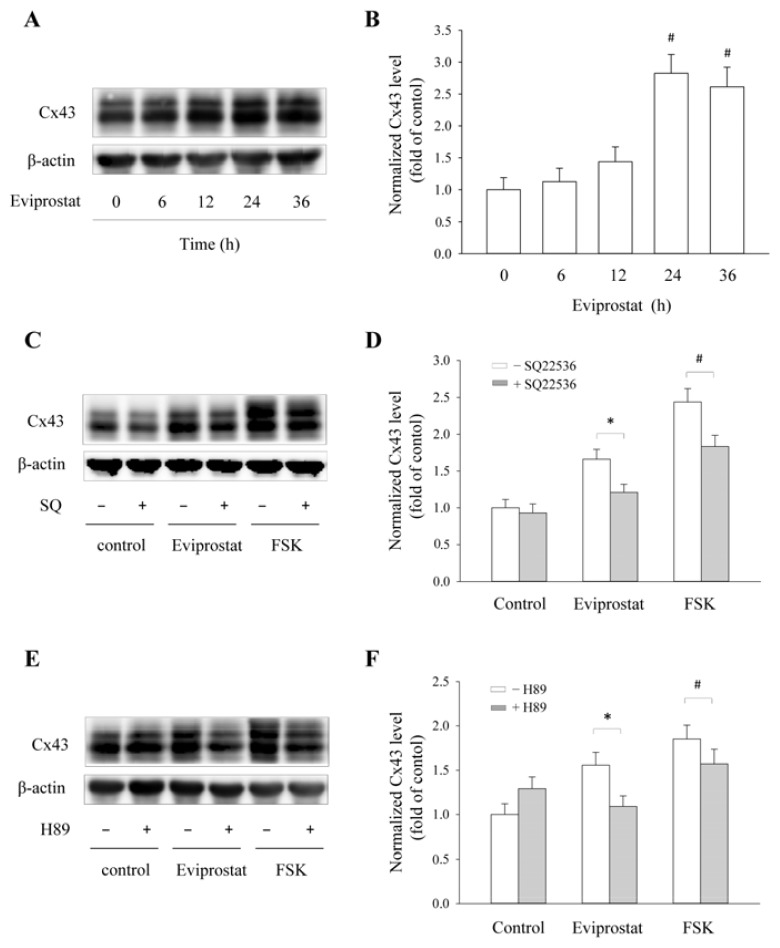
Induction of Cx43 expression by Eviprostat. (**A** and **B**) Upregulation of Cx43 induced by Eviprostat. Mesangial cells were incubated with 50 μg/mL Eviprostat or 0.1% dimethylsulfoxide as vehicle control for indicated duration. The levels of Cx43 and β-actin were determined by Western blot analysis. Densitometric analysis of Cx43 expression were shown in B (mean ± SE, *n* = 4), # *p* < 0.01 *vs.* control. (**C**–**F**) Inhibition of adenylyl cyclase (AC) and PKA inhibitors on Cx43 levels induced by Eviprostat. Mesangial cells were pretreated to 50 μM AC inhibitor SQ22536 or 10 μM H89 for 30 min before exposing to 50 μg/mL Eviprostat or 10 μM FSK for 24 h in the continuous presence of the inhibitors. Densitometric analysis of Cx43 expression of C and E were shown in **D** and **F**, respectively. The results were normalized to corresponding β-actin level, and data were presented as relative induction against the basal level. # *p* < 0.01, * *p* < 0.05.

Activation of CREB should lead to activation of genes that have CRE binding sites. To confirm this, we have determined the effect of Eviprostat on the expression of Cx43, a CRE-controlled gene product [20,21]. As shown in Figure 3A,B, incubation of mesangial cells with Eviprostat caused a time-dependent elevation in Cx43 level. This effect was abolished in the presence of adenylyl cyclase (AC) inhibitor SQ22536 (Figure 3C,D) and PKA inhibitor H89 (Figure 3E,F), whereas it was mimicked by AC activator, FSK. These observations thus indicate that Eviprostat activates the cAMP signaling pathway.

### 2.3. Eviprostat Suppresses PDGF-Induced Cellular Proliferation

**Figure 4:**
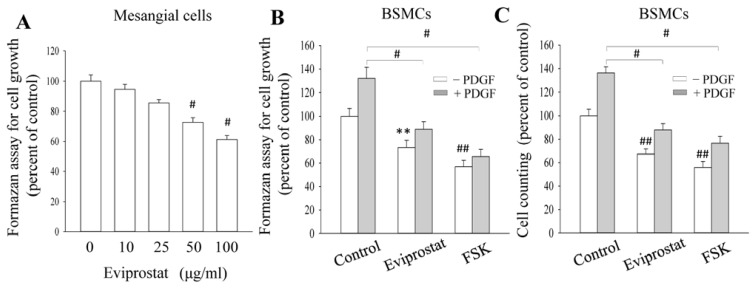

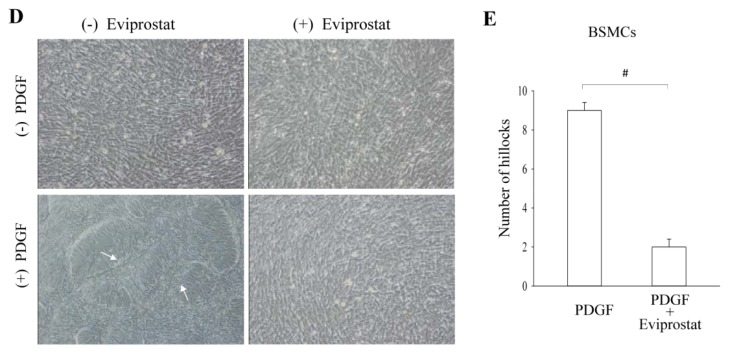
Anti-proliferative effect of Eviprostat. (**A**) Dose-dependent effects of Eviprostat on mesangial cell proliferation. Mesangial cells were pretreated with the indicated concentrations of Eviprostat for 1 h before exposing to 20 ng/mL platelet-derived growth factor (PDGF) for 24 h. Cell growth was evaluated by formazan assay. The data were expressed as percent of the control at zero point (mean ± SE, *n* = 4), # *p* < 0.01 *vs.* zero point control. (**B** and **C**) Effects of Eviprostat and forskolin (FSK) on bladder smooth muscle cells (BSMCs) proliferation. BSMCs were treated with 50 μg/mL Eviprostat or 10 μM FSK for 1 h before incubation with 20 ng/mL PDGF for an additional 24 h. Cell growth was determined by either formazan assay (**B**) or direct cell counting (**C**). The data were expressed as percent of untreated control (mean ± SE, *n* = 4); # *p* < 0.01 *vs.* PDGF alone, ** *p* < 0.05 and ## *p* < 0.01 *vs.* untreated control. (**D** and **E**) Effect of Eviprostat on hillock formation. BSMCs were pretreated with 50 μg/mL Eviprostat for 12 h or left untreated and exposed for 20 ng/mL PDGF for additional 48 h. The cell morphology was photographed (**C**, magnification: 200×), and the number of hillocks was manually counted in a blind manner and were shown in D. Results were expressed as the average number of hillocks counted in ten fields. # *p* < 0.01 *vs.* PDGF alone. Arrows denote multilayered ridges and nodules, the characterized feature of hillocks.

Several previous studies have demonstrated that cAMP suppresses cell proliferation and matrix production [22]. We therefore determined whether Eviprostat could inhibit PDGF-induced cellular proliferation. In Figure 4A, incubation of mesangial cells with Eviprostat led to concentration-dependent inhibition of PDGF-elicited mesangial cell proliferation. This significant inhibition was achieved at the concentration of 50 μg/mL. Besides glomerular mesangial cells, Eviprostat also inhibited PDGF-induced cell proliferation in BSMCs, as determined by formazan formation and direct cell counting. As a positive control, AC activator, FSK, indeed inhibited BSMC proliferation (Figure 4B,C). We also examined the effect of Eviprostat on the hillock formation, an *in vitro* model of vascular and glomerular sclerosis [23]. In Figure 4D, PDGF dramatically facilitated the formation of hillocks, characterized by multilayered ridges and nodules. In the presence of Eviprostat, this effect of PDGF was pronouncedly inhibited (Figure 4E). Eviprostat also suppressed hillock formation in glomerular mesangial cells (data not shown). These results suggest that Eviprostat inhibits both cell proliferation and matrix production.

### 2.4. Eviprostat Suppresses PDGF-Induced Activation of PI3K and MAPK

**Figure 5:**
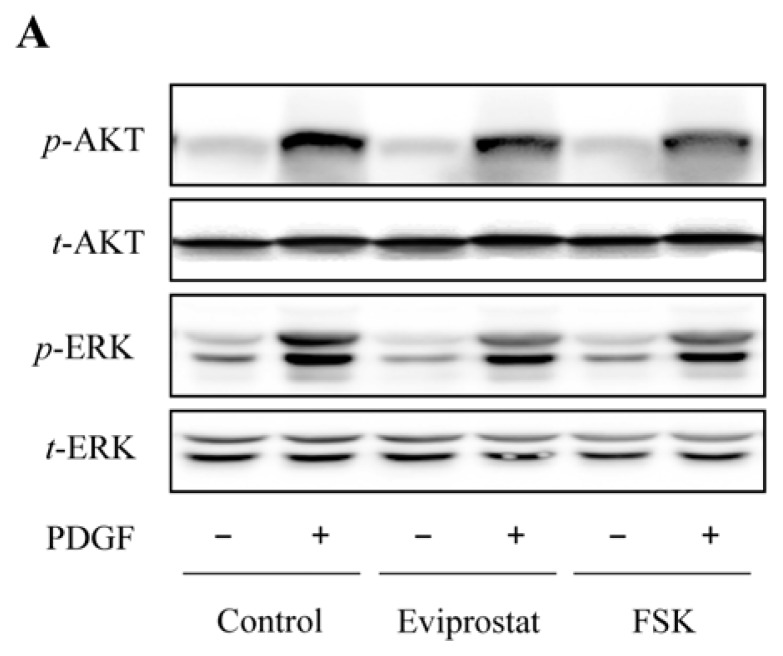

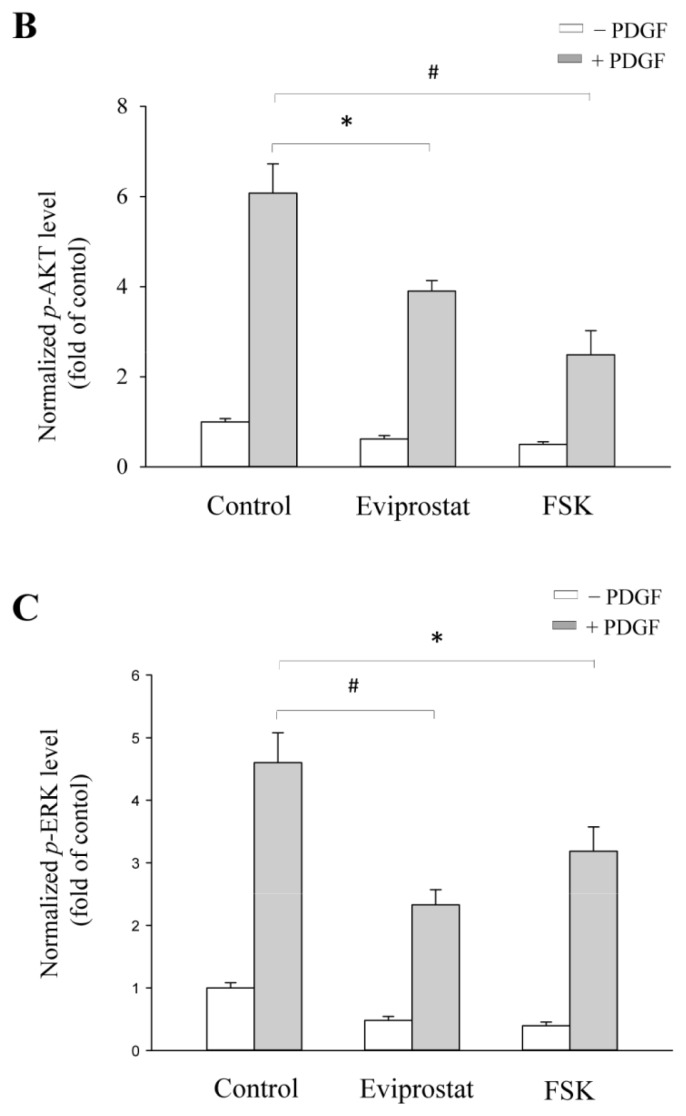
Interference of PI3K and MAPK activation by Eviprostat. (**A**) Inhibition of PDGF-induced *p*-ERK and *p*-AKT by Eviprostat. BSMCs were pretreated to 50 μg/mL Eviprostat and 10 μM FSK for 1 h before exposing to 25 ng/mL PDGF for 24 h. The control was treated with vehicle (0.1% dimethylsulfoxide). Cellular proteins were extracted. The levels of *p*-ERK and *p*-AKT were determined by Western blot analysis, and total ERK and AKT were used as internal control, respectively. (**B** and **C**) Densitometric analysis of *p*-ERK and *p*-AKT expression shown in A. The results were normalized to corresponding internal controls, and data were presented as induction relative to the basal level in control (mean ± SE, *n* = 4). # *p* < 0.01, * *p* < 0.05.

The mitogenic effect of PDGF is reported to be mediated by PI3K and MAPK signaling pathways [13,14]. We, therefore, examined the influence of Eviprostat on these kinases and cellular proliferation in BSMCs. As shown in Figure 5, Eviprostat greatly suppressed PDGF-induced phosphorylation of ERK and Akt. The inhibition effect of Eviprostat was also mimicked by AC activator, FSK. The results thus indicate that Eviprostat interferes with PDGF-induced mitogenic signaling pathway in BSMCs.

### 2.5. Eviprostat Increases Cx43 Levels via cAMP Signaling Pathway in BSMCs

**Figure 6:**
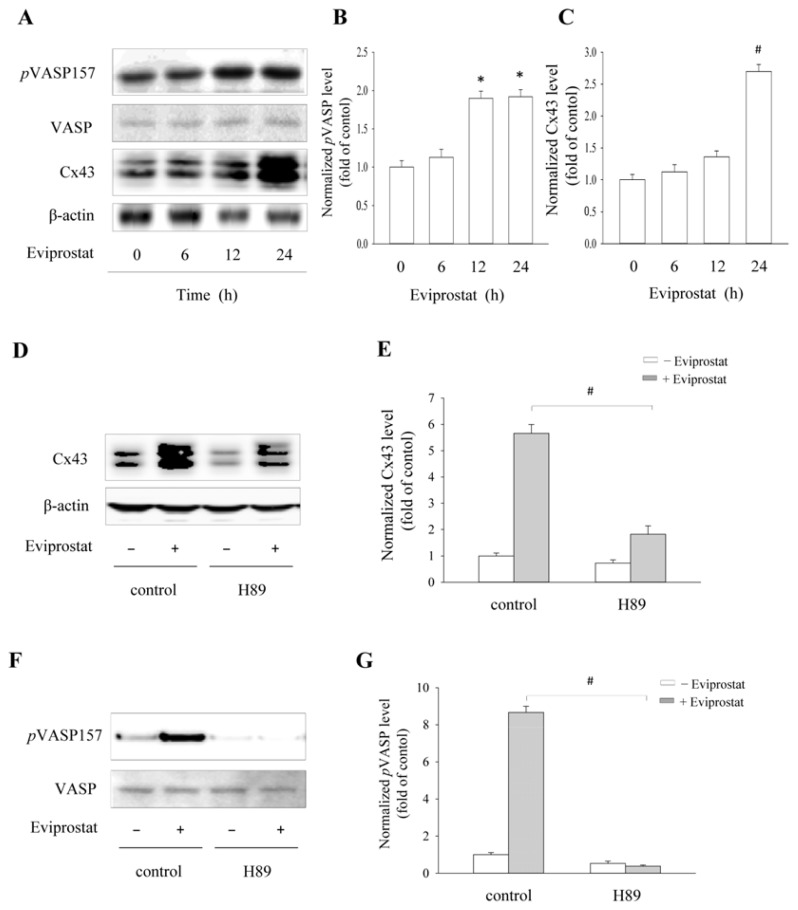
Activation of cAMP signaling pathway and induction of Cx43 by Eviprostat in BSMCs. (**A**) Time-dependent effects of Eviprostat on *p*VASP and Cx43. BSMCs were exposed to 50 μg/mL Eviprostat or 0.1% dimethylsulfoxide as vehicle control for the indicated duration. Cellular protein was extracted and subjected to Western blot analysis using specific antibodies for *p*VASP and Cx43. Equal loading of protein per lane was verified by reprobing the blot with non-phosphorylated VASP and β-actin antibody. (**B**, **C**) Densitometric analysis of *p*VASP and Cx43 in A. The results were normalized to corresponding internal controls, and data were presented as induction relative to the basal level in control. # *p* < 0.01, * *p* < 0.05. (**D**–**G**) Effect of PKA inhibitor on Cx43 levels and VASP phosphorylation induced by Eviprostat. BSMCs were pretreated with 10 μM H89 for 30 min before exposing to 50 μg/mL Eviprostat for an additional 24 h. The levels of Cx43 and pVASP were determined by Western blot analysis, and non-phosphorylated VASP and β-actin were used as internal control. Densitometric analysis of Cx43 and pVASP level in D and F were shown in E and G, respectively. # *p* < 0.01.

To further confirm the effects of Eviprostat on the cAMP pathway in BSMCs, we examined the expression of Cx43 and phosphorylated VASP in the presence of Eviprostat. As shown in Figure 6, Eviprostat induced a time-dependent increase in the level of Cx43 and VASP phosphorylation at serine 157 (Figure 6A,C). The elevation of Cx43 was largely blocked by PKA inhibitor, H89. As expected, H89 effectively prevented the phosphorylation of VASP at both basal and Eviprostat-stimulated conditions (Figure 6D,G). These data suggest that similar to mesangial cells, Eviprostat also activates the cAMP signaling pathway in BSMCs.

### 2.6. Discussion

In this study, we revealed, for the first time, that Eviprostat was able to activate the cAMP signaling pathway. Given that cAMP exerts multiple effects on cellular processes and that cAMP-elevating agents have been widely used for treatment of a variety of diseases, our finding about activation of the cAMP signaling pathway by Eviprostat could have great clinical implications.

cAMP is an important intracellular second messenger responsible for a variety of cellular responses to external stimuli [7,24]. It is raised by increased synthesis via activation of AC and/or decreased degradation via inhibition of PDEs [16–18]. Elevated cAMP causes PKA activation and subsequently activates CRE, leading to expression of genes that have CRE in their regulatory regions [25]. In this investigation, we have used a CRE-SEAP-reporting system to detect the potential influence of Eviprostat on the cAMP signaling pathway. This system, which has been successfully used for profiling of functional PDEs in mesangial cells, is a simple, sensitive and specific method for detection of alterations in the cAMP signaling cascade [16–18]. Taking advantage of this system, we observed that Eviprostat stimulated SEAP secretion, which was associated the increased phosphorylation of PKA substrates, as well as the enhanced expression of CRE-controlled gene product, Cx43 [16,21]. These observations thus pointed to an activation of the cAMP signaling pathway. Consistent with this conclusion, blockade of cAMP generation by SQ22536 or inhibition of PKA activation with H89 indeed prevented the effect of Eviprostat on Cx43 expression. This critical role of cAMP-generating adenylyl cyclase and cAMP-activated kinase PKA also indirectly indicate an involvement of the elevated cAMP in the effect of Eviprostat.

How did Eviprostat activate the cAMP signaling pathway? Our results indicated that Eviprostat might activate cAMP pathway through inhibition of PDE4 activity. This was shown by the fact that Eviprostat worked cooperatively with the PDE3 inhibitor, but not the PDE4 inhibitor, in the induction of CRE activation. In our previous studies, we have characterized PDE3 and PDE4 as two major enzymes responsible for the breakdown of cAMP in reporter mesangial cells. We have demonstrated that the cooperative activation of cAMP signaling pathway in mesangial cells could only be achieved through concomitant suppression of PDE3 and PDE4 [16–18]. In this context, the potentiating effect of Eviprostat was most likely mediated by its action on PDE4. To confirm this finding, detailed analysis of the effect of Eviprostat on PDE4 expression and activity may be needed in the future. Besides PDE4, it might also be worth testing whether Eviprostat affects the activity of adenylyl cyclase. In this investigation, we observed that inhibition of adenylyl cyclase largely prevented the effect of Eviprostat on Cx43 expression. This effect could be due to either the direct suppression of Eviprostat-induced activation of adenylyl cyclase or indirect consequence through inhibition of cAMP generation, which would weaken the effect of PDE inhibition on cAMP accumulation.

Our study also found that Eviprostat interfered with the PDGF signaling pathway and inhibited cell proliferation in both mesangial cells and BSMCs. This observation is not surprising, because anti-mitogenic effects of cAMP-elevating agents have been well described [7,8,17]. PDGF, as an important growth factor implicated in bladder injury and remolding, has been shown to play an important role in cell proliferation and matrix production [12,14,26]. In accordance with the previous reports, we demonstrated that it promoted hillock formation in BSMCs and glomerular mesangial cells. Hillock, a result of focally prominent cell proliferation and extracellular matrix accumulation, has been considered as an ideal *in vitro* model of sclerosis [23,27], inhibition of which suggests that Eviprostat could also be used for amelioration of sclerotic lesions seen in glomerulosclerosis and bladder remodeling following BOO.

Our findings could have important implications. First, we have identified a novel mechanism involved in the therapeutic effects of Eviprostat. The anti-inflammatory and anti-proliferative effects of Eviprostat could be at least partially mediated by the increased intracellular cAMP. Several reports described that cAMP/PKA inhibited NFκB function by slowing down its translocation into the nucleus and preventing gene transcription [28]. In addition, cAMP could also impact the cytokine-induced proinflammatory responses through regulation of Cx43. Our recent study revealed that Cx43 acted as a negative feedback mechanism for cytokines-induced production of nitric oxide [21]. Apart from its anti-inflammatory actions, cAMP is also known for its anti-mitogenic actions in both mesangial cells and BSMCs [6,8,12,13]. Keeping in line with the previous observations, the induction of cAMP by Eviprostat was, indeed, associated with an obvious reduction in cell growth. In addition, the effective concentration of Eviprostat used for suppression of inflammation and cell BSMCs were about 30~100 μg/mL [15], which overlaps with the concentration used in this study for activation of the cAMP signaling pathway; Second, as a cAMP-elevating agent with few side effects in clinic, Eviprostat may have a wide range of therapeutic potential. At present, the application of Eviprostat is limited to the field of lower urinary tract disorders. As a potential PDE4 inhibitor, it may be used for treatment of a wide range of diseases.

It should be noted that Eviprostat is a mixture of compounds from various natural sources. At present, the ingredients and molecular mechanisms responsible for Eviprostat-induced activation of the cAMP signaling pathway are unclear. More detailed analysis is needed to answer these questions. It is also worth testing whether other cAMP-elevating agents, especially PDE4 inhibitors, could achieve the similar therapeutic efficacy as Eviprostat in clinic.

## 3. Experimental Section

### 3.1. Reagents

The extract mixture of Eviprostat dissolved in 0.1% dimethylsulfoxide in distilled water was obtained from Nippon Shinyaku Co., Ltd. PDGF-BB was purchased from R&D Systems (Minneapolis, MN, USA). Anti-vasodilator-stimulated phosphoprotein (anti-VASP) at serine 157 was obtained from Chemicon International (Temecula, CA, USA). Cilostamide, rolipram, SQ22536, H89, cAMP, FSK, anti-Cx43 and anti-β-actin antibodies, as well as all other chemicals were obtained from Sigma (Tokyo, Japan).

### 3.2. Establishment of Reporter Cells

Clonal mesangial cells (SM43) were established from isolated renal glomeruli of a male Sprague-Dawley rat and identified as being of the mesangial cell phenotype, as described before [16–18]. Using a calcium-phosphate co-precipitation method, SM/cAMP response element (CRE)-secreted alkaline phosphatase 15 (SEAP 15) cells were established by transfection of SM43 cells with pCRE-SEAP (BD Bioscience, Palo Alto, CA, USA), as described previously [16–18]. pCRE-SEAP encodes SEAP under the control of three copies of CRE.

### 3.3. Bladder Smooth Muscle Cell Culture

BSMCs were established from the bladder of Sprague-Dawley rats, as reported previously [29].

### 3.4. Western Blot Analysis

Western blot was performed by the enhanced chemiluminescence system, as described before [13,21]. Chemiluminescent signal is quantified with densitometric software Fujifilm Image Gauge (Fujifilm, Tokyo, Japan). The results were normalized to corresponding internal controls to correct for loading. Data were presented as the fold change relative to the level in control.

### 3.5. SEAP Assay

Activity of SEAP was evaluated using the Great EscAPe Detection Kit (BD Bioscience), following the protocol provided by the manufacturer [16–18]. The intensity of chemiluminescent signal was determined by a luminometer (Gene Light 55; Microtech Nition, Chiba, Japan). Assays were performed in quadruplicate. SEAP activity was expressed as fold induction against control.

### 3.6. Formazan Assay

The cell proliferation was assessed by formazan assay using Cell Counting Kit-8 (Dojindo Laboratory, Kumamoto, Japan), as described before [17,30].

### 3.7. Cell Counting

Cells in a 96-well plate were exposed to PDGF in the presence or absence of Eviprostat or forskolin for 24 h. Then, cells were digested with 0.25% trypsin, and a blinded experimenter conducted manual cell counts using phase-contrast microscope.

### 3.8. Statistical Analysis

Values were expressed as the mean ± SE (*n* = 4). A comparison of two populations was made using Student′s *t*-test. For multiple comparisons with a single control, one-way analysis of variance (ANOVA) followed by Dunnett′s test was employed. Both analyses were carried out using SigmaStat statistical software (Systat Software, Inc., San Jose, CA, USA, 2011). *p* < 0.05 was considered to be a statistically significant difference.

## 4. Conclusions

Our study indicates that activation of the cAMP signaling pathway is one of the important mechanisms underlying the therapeutic effects of Eviprostat.

## References

[1] Berry S.J., Coffey D.S., Walsh P.C., Ewing L.L. (1984). The development of human benign prostatic hyperplasia with age. J. Urol.

[2] Lee K.L., Peehl D.M. (2004). Molecular and cellular pathogenesis of benign prostatic hyperplasia. J. Urol.

[3] Ishigooka M., Hashimoto T., Hayami S., Tomaru M., Nakada T., Mitobe K. (1995). Clinical and retrospective evaluation of Eviprostat: A non-hormonal and non-neuropharmacological agent for benign prostatic hyperplasia. Int. Urol. Nephrol.

[4] Oka M., Tachibana M., Noda K., Inoue N., Tanaka M., Kuwabara K. (2007). Relevance of anti-reactive oxygen species activity to anti-inflammatory activity of components of eviprostat, a phytotherapeutic agent for benign prostatic hyperplasia. Phytomedicine.

[5] Do Monte F.H., dos Santos J.G., Russi M., Lanziotti V.M., Leal L.K., Cunha G.M. (2004). Antinociceptive and anti-inflammatory properties of the hydroalcoholic extract of stems from *Equisetum arvense* L. in mice. Pharmacol. Res.

[6] Tasken K., Stokka A.J. (2006). The molecular machinery for cAMP-dependent immunomodulation in T-cells. Biochem. Soc. Trans.

[7] Andersson K.E., Arner A. (2004). Urinary bladder contraction and relaxation: Physiology and pathophysiology. Physiol. Rev.

[8] Misra U.K., Pizzo S.V. (2005). Coordinate regulation of forskolin-induced cellular proliferation in macrophages by protein kinase A/cAMP-response element-binding protein (CREB) and Epac1-Rap1 signaling: Effects of silencing CREB gene expression on Akt activation. J. Biol. Chem.

[9] Ritchie R.H., Rosenkranz A.C., Huynh L.P., Stephenson T., Kaye D.M., Dusting G.J. (2004). Activation of IP prostanoid receptors prevents cardiomyocyte hypertrophy via cAMP-dependent signaling. Am. J. Physiol. Heart Circ. Physiol.

[10] Lee S.R., Hong C.H., Choi Y.D., Kim J.H. (2010). Increased urinary nerve growth factor as a predictor of persistent detrusor overactivity after bladder outlet obstruction relief in a rat model. J. Urol.

[11] Monga M., Gabal-Shehab L.L., Stein P. (2001). Urinary transforming growth factor-beta1 levels correlate with bladder outlet obstruction. Int. J. Urol.

[12] Li K., Yao J., Sawada N., Kitamura M., Andersson K.E., Takeda M. (2012). β-Catenin signaling contributes to platelet derived growth factor elicited bladder smooth muscle cell contraction through up-regulation of Cx43 expression. J. Urol.

[13] Yao J., Kitamura M., Zhu Y., Meng Y., Kasai A., Hiramatsu N., Morioka T., Takeda M., Oite T. (2006). Synergistic effects of PDGF-BB and cAMP-elevating agents on expression of connexin43 in mesangial cells. Am. J. Physiol. Renal. Physiol.

[14] Adam R.M., Roth J.A., Cheng H.L., Rice D.C., Khoury J., Bauer S.B., Peters C.A., Freeman M.R. (2003). Signaling through PI3K/Akt mediates stretch and PDGF-BB-dependent DNA synthesis in bladder smooth muscle cells. J. Urol.

[15] Tagaya M., Oka M., Ueda M., Takagaki K., Tanaka M., Ohgi T., Yano J. (2009). Eviprostat suppresses proinflammatory gene expression in the prostate of rats with partial bladder-outlet obstruction: A genome-wide DNA microarray analysis. Cytokine.

[16] Yao J., Hiramatsu N., Zhu Y., Morioka T., Takeda M., Oite T., Kitamura M. (2005). Nitric oxide-mediated regulation of connexin43 expression and gap junctional intercellular communication in mesangial cells. J. Am. Soc. Nephrol.

[17] Yao J., Zhu Y., Sun W., Sawada N., Hiramatsu N., Takeda M., Kitamura M. (2007). Irsogladine maleate potentiates the effects of nitric oxide on activation of cAMP signalling pathways and suppression of mesangial cell mitogenesis. Br. J. Pharmacol.

[18] Zhu Y., Yao J., Meng Y., Kasai A., Hiramatsu N., Hayakawa K., Miida T., Takeda M., Okada M., Kitamura M. (2006). Profiling of functional phosphodiesterase in mesangial cells using a CRE-SEAP-based reporting system. Br. J. Pharmacol.

[19] Butt E., Abel K., Krieger M., Palm D., Hoppe V., Hoppe J., Walter U. (1994). cAMP- and cGMP-dependent protein kinase phosphorylation sites of the focal adhesion vasodilator-stimulated phosphoprotein (VASP) *in vitro* and in intact human platelets. J. Biol. Chem.

[20] Saez J.C., Berthoud V.M., Branes M.C., Martinez A.D., Beyer E.C. (2003). Plasma membrane channels formed by connexins: Their regulation and functions. Physiol. Rev.

[21] Li K., Yao J., Shi L., Sawada N., Chi Y., Yan Q., Matsue H., Kitamura M., Takeda M. (2011). Reciprocal regulation between proinflammatory cytokine-induced inducible NO synthase (iNOS) and connexin43 in bladder smooth muscle cells. J. Biol. Chem.

[22] Clark J.G., Kostal K.M., Marino B.A. (1982). Modulation of collagen production following bleomycin-induced pulmonary fibrosis in hamsters. Presence of a factor in lung that increases fibroblast prostaglandin E2 and cAMP and suppresses fibroblast proliferation and collagen production. J. Biol. Chem.

[23] Kitamura M. (1999). The antioxidant *N*-acetylcysteine induces mesangial cells to create three-dimensional cytoarchitecture that underlies cellular differentiation. J. Am. Soc. Nephrol.

[24] Das R., Esposito V., Abu-Abed M., Anand G.S., Taylor S.S., Melacini G. (2007). cAMP activation of PKA defines an ancient signaling mechanism. Proc. Natl. Acad. Sci. USA.

[25] Kamenetsky M., Middelhaufe S., Bank E.M., Levin L.R., Buck J., Steegborn C. (2006). Molecular details of cAMP generation in mammalian cells: A tale of two systems. J. Mol. Biol.

[26] Stehr M., Adam R.M., Khoury J., Zhuang L., Solomon K.R., Peters C.A., Freeman M.R. (2003). Platelet derived growth factor-BB is a potent mitogen for rat ureteral and human bladder smooth muscle cells: Dependence on lipid rafts for cell signaling. J. Urol.

[27] Sterzel R.B., Lovett D.H., Foellmer H.G., Perfetto M., Biemesderfer D., Kashgarian M. (1986). Mesangial cell hillocks. Nodular foci of exaggerated growth of cells and matrix in prolonged culture. Am. J. Pathol.

[28] Wall E.A., Zavzavadjian J.R., Chang M.S., Randhawa B., Zhu X., Hsueh R.C., Liu J., Driver A., Bao X.R., Sternweis P.C. (2009). Suppression of LPS-induced TNF-alpha production in macrophages by cAMP is mediated by PKA-AKAP95-p105. Sci. Signal.

[29] Sawada N., Yao J., Hiramatsu N., Hayakawa K., Araki I., Takeda M., Kitamura M. (2008). Involvement of hypoxia-triggered endoplasmic reticulum stress in outlet obstruction-induced apoptosis in the urinary bladder. Lab. Invest.

[30] Fang X., Huang T., Zhu Y., Yan Q., Chi Y., Jiang J.X., Wang P., Matsue H., Kitamura M., Yao J. (2011). Connexin43 hemichannels contribute to cadmium-induced oxidative stress and cell injury. Antioxid. Redox. Signal.

